# Magnetic resonance imaging for evaluating tumor resectability in advanced ovarian cancer: A protocol for systematic review and meta-analysis

**DOI:** 10.1097/MD.0000000000023419

**Published:** 2020-12-11

**Authors:** Ping Liu, Lingli Deng, Lijun Yang, Xianhong Yuan, Wei Xia, Yongxue Su

**Affiliations:** a Department of Radiology; b Department of Obstetrics, Maternal and Child Health Hospital of Hubei Province; c Department of Imaging Center, Wuhan Children's Hospital (Wuhan Maternal and Child Healthcare Hospital), Tongji Medical College, Huazhong University of Science and Technology, Wuhan, Hubei, P.R. China.

**Keywords:** diagnostic accuracy, magnetic resonance imaging, ovarian cancer, systematic review

## Abstract

**Background:**

This study will evaluate the diagnostic accuracy of magnetic resonance imaging (MRI) to investigate tumor resectability at primary debulking surgery among women experiencing advanced-stage ovarian cancer.

**Methods:**

We will systematically search the randomized controlled trials (RCTs) for potentially eligible studies from electronic databases, including 4 English databases (PubMed, EMBASE, Web of Science, and Cochrane Library) and 3 Chinese databases (China National Knowledge Infrastructure, WanFang, and China Biomedical Database). The study language will be restricted to English and Chinese. Also, 2 independent authors will collect and carry out data extraction as well as quality assessment. Data will be synthesized using appropriate statistical methods.

**Results:**

We will summarize present study's evidence to assess the diagnostic accuracy of MRI for evaluating tumor resectability at primary debulking surgery in women experiencing advanced-stage ovarian cancer.

**Conclusion:**

The present study will put forward the latest high-quality evidence for MRI's clinical application for evaluating tumor resectability in women experiencing advanced ovarian cancer.

**Ethics and dissemination:**

Since the present study is a systematic review and meta-analysis based on the published literature, ethical approval will not be necessary.

**Protocol registration number:**

DOI 10.17605/OSF.IO/UWDRF (https://osf.io/uwdrf/)

## 1 Introduction

Ovarian cancer is one of the leading causes of death due to gynecological cancers, with approximately 295,414 new cancer cases and 184,799 deaths worldwide in 2018.^[1]^ Because ovarian cancer often has no symptom in early stage-disease, most patients tend to be diagnosed at an advanced stage, thus the chance of 5-year overall survival rate is about 46%.^[2]^ In women with newly diagnosed epithelial ovarian cancer, the mainstream treatment is usually a blend of cytoreductive surgery with platinum-based chemotherapy.^[3]^ Despite recent improvements in the diagnosis and therapy or treatment of ovarian cancer, the projection and 5-year survival rate of ovarian cancer is still low, mainly because of diagnostic delay. Thus, early diagnosis and treatment are essential for improved outcomes. Previous studies have investigated the accuracy of magnetic resonance imaging (MRI) to evaluate tumor resectability at primary debulking surgery in women during the advanced-stage ovarian cancer.^[4–6]^ However, no systematic review has investigated the diagnostic precision of MRI for use among women experiencing advanced ovarian. This study, therefore, intends to assess the diagnostic accuracy of MRI among women experiencing advanced-stage ovarian cancer to establish its viability regarding primary debulking surgery.

## 2 Methods

The study's present procedure was registered on Open Science Framework (OSF, http://osf.io/) with a registration DOI number 10.17605/OSF.IO/UWDRF, and we have reported it under the guideline of the Preferred Reporting Items for Systematic Reviews and Meta-Analysis Protocol (PRISMA-P) Statement.^[7]^

### 2.1 Inclusion and exclusion criteria

#### 2.1.1 Inclusion criteria

The present study will incorporate a randomized controlled trials (RCTs) of MRI to evaluate tumor resectability in women with advanced-stage ovarian cancer, particularly those slated to go through primary debulking surgery. The study's languages were restricted to English and Chinese.

#### 2.1.2 Exclusion criteria

Some of the studies that will be disregarded include repeated publications, case report, letters, animal studies, and non-RCTs.

### 2.2 Types of participants

Patients above the age of 18 years met the inclusion criteria and were, therefore, considered eligible to undergo primary debulking surgery. All participants should perform MRI test.

### 2.3 Types of index tests

#### 2.3.1 Index test

MRI was applied in women experiencing advanced-stage ovarian cancer.

#### 2.3.2 Reference standards

The process of primary debulking surgery considered in the study was a reference standard. Also, the study determined the outcome category by the surgeon at the end of this surgery.

### 2.4 Types of outcome measures

The types of outcome measures included sensitivity, specificity, false negatives, and false positives.

### 2.5 Information sources and search strategy

We will systematically search RCTs for potentially eligible studies from electronic databases, including 4 English databases (PubMed, EMBASE, Web of Science, and Cochrane Library) and 3 Chinese databases (China National Knowledge Infrastructure, WanFang, and China Biomedical Database). Additionally, we will include the qualified studies published before October 18, 2020. Also, we will examine other sources, such as ClinicalTrials.gov, the reference list of all relevant studies, and grey works of literature to avoid missing potential works that can befit the present study. The following MeSH terms, related synonym, and their combinations will be searched in the databases mentioned above: “ovarian neoplasm,” “ovarian cancer,” ”ovarian tumor,” “ovarian carcinoma,” “magnetic resonance imaging,” MRI∗, “randomized controlled trial,” “randomised controlled trial,” randomly^∗^, and RCT^∗^.

### 2.6 Data collection and analysis

#### 2.6.1 Studies selection

**Figure 1:**
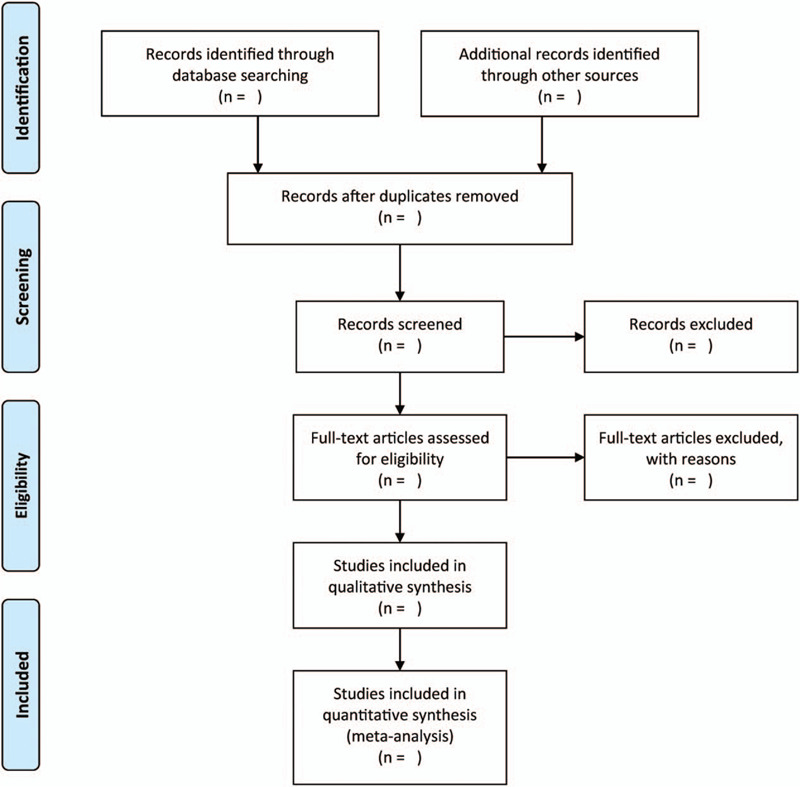
The research flowchart.

We will apply the EndNote X9 to manage all searched records and to remove duplicated publications. Two authors will independently screen titles and abstract of works of literature to eliminate irrelevant publications. Subsequently, we will assess the full-texts of all included studies to establish whether they satisfy the inclusion criteria for the present study. Accordingly, any disagreements during the process will be addressed through discussion or by consulting a third independent author where applicable. The research flowchart is shown in Fig. 1.

#### 2.6.2 Data extraction and management

Two independent authors will utilize the predesigned Excel 2019 table to mine the following information from included study: characteristics of the study: author(s), date of publication, design of the study, and sample size used in the study; characteristics of participants: their age, sex, ethnicity, tumor stage, and pathologic tumor size; outcome measurements: sensitivity, specificity, false negatives, and false positives; index tests and reference standards; and other crucial information. Accordingly, any disagreements during the process will be addressed through discussion or by consulting a third independent author where applicable.

#### 2.6.3 Assessment of methodological quality

Furthermore, we will use 2 authors to apply the Quality Assessment of Diagnostic Accuracy Studies tool-2 to assess the methodological quality.^[8]^

#### 2.6.4 Measures of treatment effect

The sensitivity and specificity measures will be estimates using the numbers of true and false negatives and positives.

#### 2.6.5 Dealing with missing data

We will obtain any missing data or unclear information from corresponding authors through email or phone.

#### 2.6.6 Assessment of heterogeneity

Additionally, *I*^2^ value will be utilized to establish the heterogeneity. When *I*^2^ < 50%, it denotes that there is no evident statistic heterogeneity. Then, a fixed-effects model will be employed to merge data^[9]^; as well as the random-effects model to merge data.^[10]^

#### 2.6.7 Sensitivity analysis

We will then remove low-quality studies or insufficient sample size studies to further evaluate the stability of our findings.

#### 2.6.8 Assessment of reporting biases

We will also perform the funnel plots to determine potential reporting biases.

## 3 Discussion

Although many studies have reported the diagnostic accuracy of MRI for evaluating tumor resectability at primary debulking surgery among women experiencing advanced-stage ovarian cancer, no systematic review has explored the diagnostic precision of MRI in performing this function systematically. Thus, the present study sets to determine the diagnostic accuracy of MRI in women experiencing advanced-stage ovarian cancer and establish its viability regarding primary debulking surgery. The results of the present study may provide meaningful insights and evidence for clinical practice.

## Author contributions

**Conceptualization:** Ping Liu, Yongxue Su.

**Data curation:** Ping Liu, Xianhong Yuan.

**Formal analysis:** Lingli Deng, Xianhong Yuan, Wei Xia.

**Funding acquisition:** Lingli Deng, Lijun Yang, Wei Xia.

**Investigation:** Ping Liu, Lingli Deng, Xianhong Yuan, Wei Xia.

**Methodology:** Ping Liu, Lingli Deng.

**Project administration:** Lijun Yang.

**Resources:** Lijun Yang, Wei Xia.

**Software:** Xianhong Yuan.

**Supervision:** Lijun Yang, Wei Xia.

**Validation:** Lijun Yang, Yongxue Su.

**Writing – original draft:** Ping Liu, Yongxue Su.

**Writing – review & editing:** Ping Liu, Yongxue Su.
